# Population attributable fraction of dietary risk factors for cancer mortality with a focus on gastrointestinal cancers in a population based cohort study

**DOI:** 10.1038/s41598-025-89183-x

**Published:** 2025-02-10

**Authors:** Marjan Moallemian Isfahani, Sahar Dalvand, Nahid Raei Dehaghi, Maryam Sharafkhah, Sadaf G. Sepanlou, Maryam Hashemian, Hossein Poustchi, Alireza Sadjadi, Gholamreza Roshandel, Masoud Khoshnia, Alireza Delavari, Negar Rezaei

**Affiliations:** 1 Liver and Pancreatobiliary Diseases Research Center, Digestive Diseases Research Institute, Tehran University of Medical Sciences, Tehran, Iran; 2 Tehran University of Medical Sciences, Tehran, Iran; 3 Epidemiology and Community Health Branch, National Heart, Lung, and Blood Institute, National Institutes of Health, Bethesda, MD USA; 4 Digestive Oncology Research Center, Digestive Diseases Research Institute, Shariati Hospital, Tehran University of Medical Sciences, Tehran, Iran; 5 Golestan Research Center of Gastroenterology and Hepatology, Golestan University of Medical Sciences, Gorgan, Iran; 6 Digestive Disease Research Center (DDRC), Digestive Disease Research Institute, Tehran University of Medical Sciences, Tehran, MD, MPH Iran

**Keywords:** Diet, Cancer, Mortality risks, Cohort study, Gastrointestinal, Population Attributable Fraction, Cancer, Nutrition, Public health

## Abstract

Diet and nutrition are critical factors influencing cancer, the second leading cause of death worldwide. This study evaluated dietary risk factors and cancer mortality. 49,773 participants aged 40–75 years from the Golestan Cohort Study (GCS) were followed for a median of 15 years. Dietary intake was assessed using a validated food frequency questionnaire. Cox proportional hazard models estimated hazard ratios (HRs) and 95% confidence intervals (CIs), while population attributable fractions (PAFs) quantified the impact of reducing dietary risk factors. Low fruit intake accounted for 4.7% (95% CI: 0.5-8.7%) of all cancer deaths and 4.91% (95% CI: 0-9.85%) of male cancer deaths. It contributed to 23.5% (95% CI: 4.7-38.59%) of pancreatic cancer mortality in both sexes and 29.36% (95% CI: 5.15-47.38%) of male pancreatic cancer deaths. Low omega-3 intake increased esophageal and gastric cancer mortality risks, with PAFs of 21.65% (95% CI: 1.14-37.9%) and 21.46% (95% CI: 2.81-36.53%), respectively. In females, low omega-3 intake accounted for 38.68% (95% CI: 4.05-60.81%) of gastric cancer deaths. Low fruit and omega-3 consumption elevated cancer mortality risk. Community- and individual-level interventions are essential to enhance nutrient intake and reduce cancer mortality.

## Introduction

According to estimates from the World Health Organization (WHO) in 2019, cancer has been recognized as the main cause of death in developed countries and the second main cause of death in developing countries^1^. Based on the estimations of the International Agency for Research on Cancer (IARC), the number of cancer cases and mortality will reach 30.2 and 16.3 million, respectively, by 2040^2^. Similarly, an investigation by the WHO showed an increase in mortality rates due to cancer among low- and middle-income countries^3^.

Data from several studies demonstrate that modifiable factors are associated with common cancers^4^. According to the World Cancer Research Fund (WCRF) third Expert Report, diet and nutrition are modifiable risk factors that can play a major role in the incidence of several cancers; for example, high intake of red meat is associated with high rates of colorectal cancer^5,6^. GI cancers are particularly influenced by dietary factors, as the digestive tract is directly exposed to nutrients, foods, and carcinogens in the diet, making it more susceptible to dietary impacts compared to other cancer sites^7,8^. Similarly, previous studies have shown that poor nutritional status may account for 30% of all cancers and gastrointestinal cancer types, for which the percentage increases to 70%^9,10^. Furthermore, research on dietary patterns and mortality shows that poor adherence to healthy eating is associated with a higher risk of cancer mortality 8–17% higher for women and 17–24% higher for men compared to those with diets more aligned with health-focused guidelines^11^. Accordingly, healthy diets rich in fruits and vegetables have been associated with a reduced risk of cancer mortality^12,13^.

To obtain better insights into the importance of dietary factors in cancer mortality, the population attributable fraction (PAF) of relevant nutritional risk factors can be considered a useful metric. PAF clarifies the collective impact of multiple risk factors on cancer rates, providing more actionable insights than single-factor studies. This approach aids public health efforts by quantifying the proportion of cancer cases that could be prevented through targeted dietary and lifestyle changes. Specifically, PAF estimates the share of cancer deaths that might be avoided if exposure to dietary risk factors is reduced or eliminated^14^. Prior research using PAF has demonstrated that in 2015, an estimated 80,110 new cancer cases in the United States were attributed to suboptimal diet, accounting for 5.2% of all new cases, with colorectal cancer being the most affected, largely due to low whole grain and dairy intake and high processed meat consumption^15^. Similarly, in 2019, an estimated 44% of all cancer deaths in the United States were attributable to potentially modifiable risk factors, with dietary factors such as low consumption of fruits, vegetables, dietary fiber, and calcium, and high consumption of red and processed meat, playing a significant role in cancer mortality^16^. While the impact of dietary factors on cancer risk has been illustrated in multiple studies, few large and well-powered studies have investigated the role of nutritional components in cancer mortality^17^. To our knowledge, except for the Global Burden of Disease (GBD) 2015 study, no previous cancer-PAF studies have comprehensively estimated the risk of death from dietary factors for all cancers^18^. Furthermore, while the GBD 2019 study examined the patterns of cancer mortality related to metabolic factors across 204 countries and regions from 1990 to 2019, the study did not specify the PAF of dietary factors in relation to cancer mortality^19^.

In this study, we measured the PAF based on the nutritional factors identified in the Golestan Cohort Study (GCS), a large scale, population-based cohort study initiated in northeastern Iran in the early 2000s with more than 15 years of follow up. GCS examines the high incidence the high incidence of esophageal cancer and explores lifestyle, environmental, genetic, and dietary factors in relation to cancer, cardiovascular diseases, and other chronic conditions^20^. Golestan residents in have unique dietary patterns that may contribute to the region’s high rates of esophageal cancer. Key factors include drinking tea at extremely high temperatures, low intake of fresh fruits and vegetables, and high consumption of pickled and salted foods^21,22^. These habits lead to lower antioxidant intake and higher salt consumption, possibly increasing cancer risks compared to other Iranian regions^22^. To our knowledge, this study is a unique large-scale, population-based cohort study, making its PAF estimates potentially usable by a wide range of stakeholders. Gastrointestinal (GI) cancers, which rank fourth worldwide in incidence, are the leading cause of cancer-related mortality in Iran, highlighting the importance of assessing the impact of dietary risk factors on GI cancer mortality in the present study^23,24^.In light of our findings, we suggest policy implications that may guide policymakers and stakeholders in addressing dietary factors as modifiable risk factors for cancer mortality. This guidance could further serve as an instrumental reference for policy formulation in this domain.

## Methods

### Study population

The current study employed data from the Golestan Cohort Study (GCS), which has been extensively described elsewhere^25^. The GCS is a prospective, population-based study conducted in Golestan Province that targets individuals aged 40–75 years from urban and rural areas. Enrollment, conducted from January 2004 through June 2008, resulted in a total of 50,045 participants^20^. For our study, we excluded participants with a cancer diagnosis at baseline (*n* = 211) and those lacking detailed information about their type of cancer (*n*= 61). Thus, the final data used for analysis came from 49,773 participants. All participants gave written informed consent, and the study received ethical approval from the Digestive Disease Research Institute at Tehran University of Medical Sciences. All the researchers conducted this study in compliance with the guidelines of the 2008 Declaration of Helsinki. Detailed information about the study protocol is available in prior publications^20^. Since its inception, the GCS cohort has been monitored through annual telephone interviews and occasional home visits to track health outcomes. For this analysis, we included 2,196 individuals who succumbed to cancer over 18 years of follow-up, encompassing all cancer types, to estimate the population attributable fraction of dietary risk factors.

### Questionnaires and data gathering

Participants were interviewed by trained personnel who collected data using structured questionnaires on demographics, medical history, lifestyle, and physical examination^20^. Dietary data were gathered using a food frequency questionnaire (FFQ) that included a detailed list of food items, capturing typical dietary patterns in a high-risk population for esophageal cancer in northern Iran. It was administered four times to 131 participants over a year, with validity assessed against twelve 24-hour dietary recalls and biomarkers like urinary nitrogen and plasma levels of key nutrients. Validity correlations were strong for total energy (0.75), carbohydrates (0.75), and proteins (0.76), while reproducibility showed high intraclass correlations (0.66 to 0.89), confirming the FFQ’s reliability in measuring habitual intake^26^. Reliability was tested using interclass correlation coefficients between four FFQ results collected from the same participants^26^.

### Dietary factor selection

The dietary risk factors estimated in the GCS included fruits, vegetables, legumes, red meat, omega-3 fatty acids, and total dairy products according to the World Cancer Research Fund (WCRF)^27,28^. We defined cutoff points for each dietary factor based on the WCRF as a reference^29^. The following dietary factor ranges were taken into consideration as risk factors in our analysis: fruit and vegetables < 100 g per day, legumes < 28 g per day, fibers < 25 g per day, omega-3 fatty acids < 250 mg, dairy products < 400 g, and red meat > 27 g per day.

### Assessment of the outcomes

Annually, each participant was monitored through telephone communications and in-home assessments from 2004 to November 2022. The attrition rate was less than 1%, reflecting an exceptional retention rate in this cohort study. The primary outcome in our study was cancer-related death. This identification was achieved through active follow-up and by cross-referencing cohort members with the Golestan Population-based Cancer Registry database. The information on cancer mortality was accessed from the GCS database^30^. In summary, any reported deaths were also followed by a physician on the follow-up team, who used a verbal autopsy questionnaire validated for this population by interviewing the closest relative of the deceased^30^. At the same time, the follow-up team actively collected death certificates and all available medical documents^31^. Cancer types were categorized according to the International Classification of Diseases, Tenth Revision (ICD-10). In our analysis, we assessed all cancer deaths and deaths from four specific gastrointestinal cancers: gastric (C16), pancreatic (C25), esophageal (C15), and colorectal (C18–C21).

### Statistical analysis

The hazard ratios (HRs) for dietary factors in relation to all-cancer mortality and specific GI cancer mortality were calculated using Cox proportional hazards regression models. These models also yielded associated 95% confidence intervals (CIs). The start date was the enrollment date, while the end date was defined as the date of cancer-related death, loss to follow-up, or the final day of study follow-up on January 31st, 2023, whichever occurred first. Cancer-related deaths were considered a failure. The associations between the consumption of food items, including vegetables, fruits, dairy products, red meat, legumes, and omega-3 products, and cancer mortality risk were examined. Adjustments were made for age, sex, education, socioeconomic status (SES), residence (rural/urban), smoking habits, opium use, body mass index (BMI), physical activity and energy intake. Initial adjustments also included the healthy eating index (HEI) and the Dietary Approach to Stop Hypertension (DASH) scores and have been calculated in the previous GCS analysis by Hashemian et al.^32^. These two factors did not produce meaningfully different results and were subsequently removed from the models. Additionally, the initial two years of observation were excluded to mitigate the risk of reverse causality. Categorical variables are presented as absolute and relative frequencies, and quantitative variables are presented as the means with their standard deviation (SD). The intake of dietary factors is typically reported as median values with their interquartile range (IQR). All necessary assumptions of the Cox regression model, including the proportional hazards assumption verified using the Schoenfeld residuals test, were assessed and met.

To estimate the proportion of cancer deaths attributable to each dietary factor, we calculated PAFs^33,34^. The PAFs were calculated using the following equation:

$$\:\text{P}\text{A}\text{F}=\:\:\frac{p(HR\text{a}\text{d}\text{j}-1)}{1+p(HR\text{a}\text{d}\text{j}-1)}$$

HRadj is the hazard ratio (HR) adjusted, and “P” is the prevalence of exposure to the risk factors among all populations.

The PAF is the percentage of disease-related mortality if the specific factor is omitted from the population^35^. PAF values are reported with 95% CIs. Along with our adjusted HR calculations, PAFs and 95% CIs for dietary risk factors were calculated for total and GI cancers and separately by sex, other potential variables have shown no significant results. P values less than 0.05 were considered to indicate statistical significance. All of the statistical analyses in this study were carried out using STATA v.12.

## Results

A total of 50,045 participants were enrolled in the GCS. During a median 15-year follow-up of cohort participants, the total number of cancer deaths was 2,196. Most of the cancer deaths occurred at a mean ± SD of 57.37 ± 9.3 years, and most were male (56.79%). Among all cancer deaths, the largest proportion were due to gastrointestinal cancers, with 1099 (50.04%) deaths. Participants who died of cancer tended to live in rural areas and had lower wealth scores (Table 1).

**Table 1:** Selected characteristics of Golestan Cohort Study participants.

	Person years	Non-cases (*n* = 47,578)	Cancer death cases (*n* = 2196)
**Mean Age (years)**		51.79 ± 8.8	57.37 ± 9.3
**Sex**,** n (%)**			
Female	414226.06	27,696 (58.21%)	949 (43.21%)
Male	289483.5	19,882 (41.79%)	1,247 (56.79%)
**Residence**,** n (%)**			
Urban	557738.95	9,538 (20.05%)	397 (18.08%)
Rural	145970.61	38,040 (79.95%)	1,799 (81.92%)
**Education**,** n (%)**			
Illiterate	486473.72	33,253 (69.89%)	1,685 (76.73%)
Educated	217235.84	14,325 (30.11%)	511 (23.27%)
**SES**,** n (%)**			
First tertile	245900.04	16,870 (35.46%)	941 (42.85%)
Second tertile	217562.33	14,710 (30.92%)	688 (31.33%)
Third tertile	240247.19	15,998 (33.62%)	567 (25.82%)
**BMI cat**			
18.5 to 24.9 kg/m^2	224671.31	15,229 (32.01%)	856 (38.98%)
Under 18.5 kg/m^2	54384.4	3,874 (8.14%)	274 (12.48%)
25 to 29.9 kg/m^2	241388.93	16,246 (34.15%)	632 (28.78%)
Greater than or equal to 30 kg/m^2	183264.92	12,229 (25.70%)	434 (19.76%)
**Diabetes**			
Yes	43956.49	3,338 (7.02%)	176 (8.01%)
No	659753.07	44,240 (92.98%)	2,020 (91.99%)
**HTN**			
Yes	269176.3	19,014 (39.96%)	946 (43.08%)
No	434533.26	28,564 (60.04%)	1,250 (56.92%)
**Dietary factors**			
**Fruit**			
Median intake (IQR), g/day		118.68 (70.34–192.42)	110.04 (63.94- 188.15)
Low intake, n (%)	290991.96	19,997 (42.03%)	1,028 (46.81%)
**Vegetable**			
Median intake (IQR), g/day		172.46 (128.31–225.7)	170.04 (125.97–220.42)
Low intake, n (%)	99471.06	6,853 (14.40%)	365 (16.62%)
**Red meat**			
Median intake (IQR), g/day		10.93 (5–20.6)	11.09 (4.85–21.27)
High intake, n (%)	111063.62	7,332 (15.41%)	344 (15.66%)
**Legumes**			
Median legumes intake (IQR), g/day		12.22 (7.22–18.73)	12 (6.95–18.79)
Low intake, n (%)	633375.35	42,861 (91.67%)	1,955 (91.36%)
**Fiber**			
Median intake (IQR), g/day		22.49 (17.91–26.68)	22.41 (17.58–26.74)
Low intake, n (%)	450395.51	30,849 (65.96%)	1,407 (65.75%)
**Omega-3**			
Median intake (IQR), g/day		115.26 (65.55- 187.11)	109 (57.93- 178.56)
Low intake, n (%)	540925.23	36,477 (78%)	1,711 (79.95%)
**Dairy products**			
Median intake (IQR), g/day		167.67 (96.49–260.81)	164.31 (90.6–263.8)
Low intake, n (%)	651497.99	44,167 (92.83%)	2,019 (91.94%)

Data are Mean ± SD, Median (IQR) or number (%). Percentages were calculated on the basis of participants with available data. Low intakes of dietary factors including fruits, vegetables, red meat, legumes, fiber, omega-3, and dairy products were defined as intake less than the reference amount recommended by the World Cancer Research Fund (WCRF).

Abbreviations: N, number; SES, socioeconomic status.

In both sexes combined, low fruit intake had a significant hazard ratio (HR) of 1.11 (95% CI: 1.01–1.21) and contributed to 4.7% (95% CI: 0.5–8.7%) of all cancer deaths. No significant associations were detected between low vegetable intake, low omega-3 consumption, low fiber intake, or high red meat consumption and total cancer mortality. (Table 2).

**Table 2:** Associations of dietary factors with cancers mortality and their estimated PAFs (%), 95% confidence interval.

	HR (95% CI)	*P* Value	PAF
	Total		Total
Low fruits intake < 100 g per day			
All cancers	1.11 (1.01–1.21)	**0.02**	**4.7 (0.5–8.7)**
Gastric cancer	1.13(0.92–1.39)	0.23	5.52 (−3.99-14.16)
Pancreatic cancer	1.74 (1.15–2.64)	**0.008**	**23.5 (4.7–38.59)**
Esophageal cancer	1.08 (0.87–1.32)	0.45	3.96 (−7.03-13.83)
Colorectal cancer	1.12 (0.78–1.61)	0.52	4.6 (−10.62-17.73)
**Low vegetable intake** **< 100 g per day**			
All cancers	0.99 (0.87–1.12)	0.89	−0.1 (−2.07-1.77)
Gastric cancer	0.94 (0.71–1.25)	0.69	−0.89 (−5.37-3.39)
Pancreatic cancer	1.17(0.69–1.99)	0.55	2.5 (−6.65-10.87)
Esophageal cancer	0.93 (0.7–1.22)	0.62	−1.18 (−5.94-3.34)
Colorectal cancer	0.82 (0.48–1.42)	0.49	−2.42 (−9.08-3.82)
**High red meat intake** **> 27 g per day**			
All cancers	1.08 (0.65–1.21)	0.19	1.15 (−0.6-2.91)
Gastric cancer	1.13 (0.87–1.47)	0.33	2 (−2.29-6.11)
Pancreatic cancer	0.91 (0.52–1.60)	0.74	−1.34 (−9.48-6.18)
Esophageal cancer	1.19 (0.9–1.57)	0.22	2.31 (−1.68-6.16)
Colorectal cancer	0.74 (0.45–1.21)	0.23	−4.4 (−11.17-1.95)
**Low legumes intake** **< 28 g per day**			
All cancers	1.01 (0.87–1.18)	0.8	1.76 (−12.89-14.52)
Gastric cancer	1.46 (0.96–2.21)	0.07	29.75 (−3.85-52.49)
Pancreatic cancer	1.78 (0.78–4.11)	0.17	41.54 ( −27.84-73.27)
Esophageal cancer	1.14 (0.78–1.66)	0.48	11.5 (−25.05-37.36)
Colorectal cancer	0.77 (0.46–1.3)	0.33	−25.29 (−97.3-20.32)
**Low fiber intake** **< 25 g per day**			
All cancers	1.01 (0.92–1.11)	0.77	0.92 (−5.7-7.15)
Gastric cancer	0.96 (0.77–1.21)	0.78	−2.08 (−18.46-12.02)
Pancreatic cancer	1.18 (0.76–1.83)	0.44	11.11 ( −20.81- 34.6)
Esophageal cancer	0.81 (0.63–1.03)	0.09	−15.61 (−36.53-2.1)
Colorectal cancer	1.07 (0.75–1.54)	0.69	4.57 (−20.73-24.57)
**Low omega-3 intake** **< 250 mg**			
All cancers	1.1 (0.98–1.22)	0.08	7.28 (−1.05-14.93)
Gastric cancer	1.35 (1.04–1.75)	**0.02**	**21.46 (2.81–36.53)**
Pancreatic cancer	0.81 (0.52–1.27)	0.37	−16.58 (−61.79-15.99)
Esophageal cancer	1.34 (1.02–1.77)	**0.03**	**21.65 (1.14–37.9)**
Colorectal cancer	1.03 (0.69–1.54)	0.85	2.78 (−31.05-27.88)
**Low dairy products intake** **< 400 g per day**			
All cancers	0.94 (0.81–1.11)	0.51	−4.96 (−21.29-9.15)
Gastric cancer	1 (0.7–1.43)	0.97	0.49 (−38.41-28.46)
Pancreatic cancer	0.87(0.44–1.69)	0.68	−13.31 (−107.56-38.13)
Esophageal cancer	0.72 (0.5–1.02)	0.06	−36.28 (−88.53-1.47)
Colorectal cancer	1.4 (0.73–2.7)	0.30	27.02 (−33.92-60.23)

High intakes were used as the reference except for red meat where low intake was used as the reference. CI, confidence interval; HR, hazard ratio. Adjusted for age, gender, education, socioeconomic status (SES), residence (rural/urban), smoking habits, opium use, energy intake, Body Mass Index (BMI), and Physical Activity. PAF, population attributable fraction.

Most of the participants (approximately 93%) were likely to have a suboptimal intake of dairy products. Despite the high incidence of low dairy intake in the population, it was not associated with total cancer mortality (HR = 0.94, 95% CI = 0.81–1.11). Stratifying the data by sex, a significant association was observed between low fruit intake and total cancer deaths in males, with an HR of 1.12 (95% CI: 1–1.27). Among males, those with low fruit intake had a PAF of 4.91% (95% CI = 0–9.85%). However, no significant association was found between overall cancer mortality and low fruit intake in females (HR = 1.09, 95% CI = 0.95–1.25).

Regarding gastrointestinal (GI) cancers, low fruit intake was significantly associated with pancreatic cancer mortality in both sexes, with an HR of 1.74 (95% CI = 1.15–2.64), accounting for 23.5% (95% CI = 4.7–38.59%) of total pancreatic cancer deaths. Furthermore, in the sex-stratified analysis for each GI cancer, males with low fruit consumption had a significantly greater risk of pancreatic cancer mortality, with an HR of 2.18 (95% CI: 1.22–3.87), contributing to 29.36% (95% CI: 5.15–47.38%) of pancreatic cancer deaths. However, no significant association was found between low fruit intake and pancreatic cancer mortality in women. Additionally, low omega-3 intake was associated with an increased risk of esophageal cancer mortality (HR = 1.34, 95% CI = 1.02–1.77), with 21.65% (95% CI = 1.14–37.9%) of esophageal cancer deaths attributable to low omega-3 intake in both sexes. Moreover, low omega-3 fatty acid intake was shown to be associated with Gastric cancer mortality (HR = 1.35, 95% CI = 1.04–1.75) and contributed to 21.46% of the total mortality (95% CI = 2.81- 36.53%). This association was also noted in females with Gastric cancer (HR = 1.81, 95% CI = 1.07–3.05), with a PAF of 38.68% (95% CI = 4.05. %−60.81%). Like estimates for total cancer mortality, a high intake of red meat as well as low vegetable, low legume, low fiber, or low dairy production were not significantly associated with GI cancer mortality (Table 3).

**Table 3:** Associations of dietary factors with cancers mortality and their estimated PAFs (%), 95% confidence interval: stratified by sex.

	HR (95% CI)	*P* Value	PAF	HR (95% CI)	*P* Value	PAF
	Female		Female	Male		Male
Low fruits intake < 100 g per day						
All cancers	1.09 (0.95–1.25)	0.21	4.39 (−2.68-10.98)	1.12 (1- 1.27)	**0.05**	**4.91(0–9.85)**
Gastric cancer	1.15 (0.79–1.67)	0.46	7.08 (−13.65-24.03)	1.12 (0.88- 1.44)	0.33	5.07 (−5.8-14.82)
Pancreatic cancer	1.37 (0.75–2.48)	0.29	15.42 (−17.85-39.31)	2.18 (1.22–3.87)	**0.007**	**29.36 (5.15–47.38)**
Esophageal cancer	0.99 (0.73–1.36)	0.99	−0.01 (−19.02-15.96)	1.15 (0.87–1.52)	0.29	6.89 (−6.95-18.95)
Colorectal cancer	1.11 (0.66–1.86)	0.69	5.02 (−22.88-26.6)	1.1 (0.65–1.84)	0.71	3.17 (−15.35-18.72)
**Low vegetable intake** **< 100 g per day**						
All cancers	0.98 (0.82–1.19)	0.9	−0.1 (−3.22-2.77)	0.99 (0.84–1.17)	0.97	−0.04(−2.59-2.44)
Gastric cancer	1.23 (0.77–1.96)	0.36	3.93 (−5.63-12.64)	0.82 (0.57–1.17)	0.27	−2.92 (−7.98-1.89)
Pancreatic cancer	0.62 (0.24–1.6)	0.32	−6.31 (−17.63-3.92)	1.8 (0.93–3.48)	0.08	10.13 (−4.1-22.42)
Esophageal cancer	1.07 (0.72–1.57)	0.73	1.29 (−6.55-8.57)	0.82 (0.55–1.22)	0.34	−2.97 (−8.97-2.68)
Colorectal cancer	0.71 (0.31–1.61)	0.41	−4.66 (−15.29-4.98)	0.91 (0.43–1.92)	0.82	−1.02 (−10.11-7.31)
**High red meat intake** **> 27 g per day**						
All cancers	1.08 (0.88–1.32)	0.41	0.91 (−1.39-3.18)	1.08 (0.93–1.25)	0.28	1.36 (−1.23-3.89)
Gastric cancer	1.26 (0.74–2.16)	0.39	2.56 (−4.06-8.77)	1.09 (0.80–1.47)	0.56	1.53 (−3.90-6.68)
Pancreatic cancer	0.68 (0.24–1.92)	0.47	−3.59 ( −12.37-4.5)	1.06 (0.53–2.11)	0.86	1.1 (−12.47-13-03)
Esophageal cancer	1.05 (0.64–1.73)	0.82	0.51 (−4.27-5.08)	1.25 (0.89–1.76)	0.19	3.69 (−2.4-9.43)
Colorectal cancer	0.73 (0.31–1.72)	0.48	−3.08 (−11.09-4.34)	0.74 (0.4–1.36)	0.33	−5.44 (−16.18-4.29)
**Low legumes intake** **< 28 g per day**						
All cancers	0.85 (0.68–1.05)	0.13	−15.61 (−39.62-4.26)	1.21 (0.97–1.51)	0.08	16.34 (−2.74-31.89)
Gastric cancer	1.62 (0.74–3.5)	0.22	36 (−32.06-68.98)	1.39 (0.85–2.28)	0.19	26.3 (−17.3-53.69)
Pancreatic cancer	1.29 (0.45–3.62)	0.62	20.62 (−104.75-69.23)	2.83 (0.68–11.76)	0.15	62.31 (−48.46-90.43)
Esophageal cancer	0.86 (0.53–1.39)	0.53	−14.82 (−76.33-25.23)	1.61 (0.87–2.96)	0.12	36.02 (−13.99-64.09)
Colorectal cancer	0.6 (0.3–1.21)	0.15	−54.37 (−177.19-14.03)	1 (0.46–2.21)	0.98	0.61 (−103.37-51.43)
**Low fiber intake** **< 25 g per day**						
All cancers	0.94 (0.8–1.1)	0.49	−4.33 (−17.79-7.58)	1.04 (0.92–1.18)	0.43	2.9 (−4.6-9.87)
Gastric cancer	1.27 (0.79–2.04)	0.31	17.72 (−20.75-43.94)	0.89(0.67–1.18)	0.44	−6.75 (−26.05-9.58)
Pancreatic cancer	1.15 (0.57–2.30)	0.69	10.45 (−54.34-48.04)	1.20 (0.67–2.17)	0.53	10.82 (−29.52-38.61)
Esophageal cancer	0.71 (0.48–1.06)	0.09	−29.95 (−76.17-4.14)	0.87 (0.64–1.18)	0.37	−8.91 (−31.07-9.5)
Colorectal cancer	0.91 (0.49–1.71)	0.79	−6.54 (−69.72-33.11)	1.16 (0.72–1.85)	0.53	8.2 (−20.86-30.27)
**Low omega-3 intake** **< 250 mg**						
All cancers	1.07 (0.91–1.27)	0.38	5.73 (−7.63-17.44)	1.11 (0.96–1.29)	0.12	8.47 (−2.54-18.3)
Gastric cancer	1.81 (1.07–3.05)	**0.02**	**38.68 (4.05–60.81)**	1.21 (0.89–1.64)	0.2	14.34 (−9.15-32.79)
Pancreatic cancer	0.62 (0.33–1.15)	0.13	−42.81 (−121.05-7.73)	1.07 (0.56–2.05)	0.82	5.33 (−55.77-42.46)
Esophageal cancer	1.36 (0.89–2.06)	0.14	22.5 (−10.15-45.48)	1.32 (0.91–1.92)	0.13	20.84 (−8.05-42.02)
Colorectal cancer	1.26 (0.66–2.39)	0.47	16.92 (−39.86-50.65)	0.9 (0.54–1.51)	0.71	−7.04 (−53.8-25.49)
**Low dairy products intake** **< 400 g per day**						
All cancers	0.84 (0.64–1.09)	0.2	−17.75 (−51.01-8.18)	1 (0.82–1.21)	0.99	0.11 (−19.24- 16.32)
Gastric cancer	0.93 (0.42–2.03)	0.86	−6.86 (−123.74-48.96)	1.01 (0.67–1.51)	0.95	1.06 (−42.91-31.5)
Pancreatic cancer	1.47 (0.35–6.14)	0.59	30.8 (−173.22-82.47)	0.69 (0.31–1.52)	0.35	−38.54 (−174.32-30.03)
Esophageal cancer	0.54 (0.31–0.94)	0.03	−78.88 (−200.35- −6.54)	0.82 (0.52–1.29)	0.4	−19.49 (−80.98-21.1)
Colorectal cancer	4.6 (0.63–33.49)	0.13	77.18 (−61.2- 96.77)	1.03 (0.51–2.10)	0.91	3.18 (−80.82-48.17)

High intakes were used as the reference except for red meat where low intake was used as the reference. CI, confidence interval; HR, hazard ratio. Adjusted for age, gender, education, socioeconomic status (SES), residence (rural/urban), smoking habits, opium use, energy intake, Body Mass Index (BMI), and Physical Activity. PAF, population attributable fraction.

## Discussion

This study specifically assessed the impact of dietary factors on the mortality of individuals with all cancers and GI cancers. The findings of this study indicate that modifying fruit intake to exceed 100 g/d could prevent 4.7% of total cancer deaths. Among gastrointestinal cancers, our results demonstrated that increasing fruit consumption can also potentially lead to a 23.5% reduction in pancreatic cancer mortality. Based on these findings, low omega-3 consumption may contribute to 21.65% of esophageal cancer deaths. Moreover, the results suggest that strategies aimed at increasing omega-3 intake above 250 mg/day may have beneficial effects, leading to a 21.46% reduction in mortality due to gastric cancer and a 38.68% reduction in female gastric cancer mortality.

Several studies have investigated the combined consumption of fruits and vegetables. In this context, the results of studies such as the European Prospective Investigation into Cancer and Nutrition (EPIC) study have consistently demonstrated an inverse relationship between the intake of fruits and vegetables and cancer-related mortality^36^. In the same line, a study by Islami et al. indicated that increasing fruit and vegetable consumption can lead to a 17.5% reduction in cancer deaths^37^. Despite these findings, only a few studies have individually explored the effects of fruit or vegetable intake on cancer mortality. Although our study did not observe a direct association between suboptimal vegetable intake and cancer mortality, certain types of vegetables, such as cruciferous and green‒yellow vegetables, showed a significant inverse association with total cancer mortality^38^. The Third Expert Report on Diet, Nutrition, Physical Activity, and Cancer, released by the WCRF and the American Institute for Cancer Research (AICR), indicates that consuming more than 200 g of fruits daily is linked with decreased cancer mortality rates^29^. These protective effects might be attributed to the high concentrations of antioxidants, notably vitamin C, in fruits, which potentially play a pivotal role in reducing cancer mortality^39^. Nonetheless, given our focus on pinpointing the lowest consumption threshold that could present a risk, the same report suggests that an intake of 100 g or less of fruits may increase cancer risk^29^. Since this intake is in line with the consumption of alcohol in our community, we selected this as our risk factor cutoff point. Our present findings further underscore the association between increased fruit intake and cancer among men. However, a study conducted by Leenders et al. revealed an inverse association between fruit consumption and the risk of cancer-related death among females^36^. Furthermore, prior studies have yielded mixed outcomes regarding any sex-specific disparities in the relationship between fruit intake and cancer mortality^40,41^. Interestingly, our results strengthen the existing evidence for increased fruit intake among males with pancreatic cancer, suggesting the potential to prevent a noteworthy 29.36% of deaths within this subgroup. Notably, a prospective cohort study conducted in Japan also aligned with our findings, revealing an overall inverse correlation between fruit intake and pancreatic cancer risk^42^. Moreover, this Japanese cohort study identified a significant risk reduction among men with the highest fruit intake frequency compared to those with the lowest frequency^43^. In contrast to our findings, earlier cohort studies have reported no substantial evidence supporting an association between fruit consumption and pancreatic cancer mortality^44–46^.

We found no association between legume intake and overall cancer mortality. This finding is consistent with that of a meta-analysis of cohort studies by Zargarzadeh et al., which also revealed no correlation between an increase of 50 g/day in legume consumption and cancer mortality^47^. However, another study focusing on the same cohort in Iran indicated a notable inverse relationship between elevated legume intake and overall cancer mortality. While both our research and this prior study share a similar direction, the distinction in their significance can be traced back to variations in the duration of follow-up periods, with an additional 6 years in our study, the inclusion of more subjects in our analysis, and differing quantile thresholds of legume consumption used to deduce the association^48^.

While the findings of a systematic review and meta-analysis of prospective studies indicate a positive effect of high red meat intake on overall cancer mortality^49^, our study revealed no significant association. In a study conducted by Rohrmann et al., findings similar to ours were observed, with no significant association identified between high red meat consumption and cancer deaths^50^. Given the inequity in red meat consumption, a universal recommendation for its intake is inappropriate. Contextual sensitivity is essential, particularly in lower-middle-income countries where the majority of the population may not have the means to afford the suggested consumption levels. This contrasts with situations in high-income countries such as the United States, where the average consumption of red meat is 100 g per day^51^. Notably, various studies associate an average red meat consumption of approximately 40 g per day with mortality and cancer risk^52^. In contrast, the median intake in our study population was only 10 g per day. Such considerations warrant careful attention from care providers and policymakers, ensuring that broad dietary recommendations are adapted to accommodate these nuanced dissimilarities.

The evidence on the association between low intake of dairy products and cancer mortality is mixed. Our findings regarding dairy intake are consistent with a meta-analysis that found no association with total cancer mortality^53^. A systematic review and meta-analysis conducted by Lu W. et al. revealed an inverse association between a low intake of total dairy products and cancer-related death. In contrast, high intake of total dairy products did not show a similar inverse association^54^. Dairy products contain a variety of bioactive compounds. These compounds play a role in the causal cycle and could have differing effects on cancer risk and mortality, which need to be considered.

Like in our analysis, evidence suggests that omega-3 fatty acids have no clear effect on overall cancer mortality^55,56^. Additionally, a study by Zhang et al. using data from NHANES 2003–2004 and 2011–2012 revealed little to no effect of omega-3 fatty acids on cancer-related deaths^57^. Among the gastrointestinal cancers in our study, gastric and esophageal cancer mortality seemed to be inversely associated with omega-3 consumption. Considering the pivotal role of chronic inflammation in cancer progression, omega-3 fatty acids may exhibit protective effects against this disease through their anti-inflammatory and immunoregulatory effects^58^. However, the number of studies examining omega-3’s association with specific GI cancer types is limited, underscoring the need for further investigation.

### Policy implications

In low-middle-income countries, food insecurity remains a pervasive challenge with broad implications for public health, economic stability, and social well-being. A significant concern in these regions is the insufficient intake of diverse dietary components, which are essential for preventing chronic diseases and promoting overall health. As the aforementioned results indicate, a low intake of dietary factors, such as fruits and omega-3 fatty acids, can contribute to cancer mortality rates. Thus, addressing this issue and implementing effective strategies are paramount not only for the health of individual populations but also for achieving global development and sustainability goals. The role of public health and policy implications in promoting healthier dietary behaviors is evident. It is imperative to prioritize the encouragement of individuals to make informed about healthy choices in line with evidence-based recommendations^29^. This can be achieved through comprehensive education campaigns and enhanced access to educational resources. Awareness and education are vital for fostering healthy eating habits and reducing the risk of cancer mortality. Understanding the challenges individuals face, such as limited capacity for behavioral changes and socioeconomic obstacles, is essential for adopting healthier diets^13,59^. To address these challenges, governments and societies must take proactive measures to support public health initiatives. It is important to highlight that these educational endeavors would be most effective if rolled out across three key strata—policymakers, healthcare professionals, and the general population—ensuring that advice and interventions are fine-tuned to fit the socioeconomic background. This approach calls for the creation of environments that facilitate healthy dietary behaviors, the implementation of policies aligned with global sustainable development goals, and the provision of widespread access to educational resources. By investing in public health and emphasizing long-term preventive measures, governments can contribute to the establishment of healthier food environments, improved dietary choices, improved food insecurity, and significant reductions in the societal burden of cancer.

This study has several strengths, including its large sample size, long follow-up period with minimal loss to follow-up, and the inclusion of a diverse range of dietary factors. However, it is important to acknowledge certain limitations. Collecting accurate information on dietary intake is challenging due to the complex and modifiable nature of the diet. The use of the FFQ to capture habitual dietary intake, while cost-effective, may not fully represent participants’ usual food choices or portion sizes and may be susceptible to recall bias.

Such bias can lead to the misclassification of dietary exposures, potentially shifting the results toward null values. Additionally, the study assumed independence among dietary factors due to the lack of strong estimates for potential interactions, which may oversimplify the effects of combined dietary exposures on outcomes. There are several methods for calculating PAF, and we applied only one; others could be explored in future studies^60–62^. Another limitation is the small sample size for certain cancers, which resulted in wide confidence intervals. However, due to the long-term follow-up of the cohort and the low incidence of cancer, this population-based statistic remains valuable for stakeholders, particularly in low- and middle-income countries where high-quality data is scarce. Future studies with larger sample sizes and more detailed dietary assessments are warranted to build upon the evidence provided by this study.

## Conclusion

The findings of this study highlight the positive association between low fruit intake and cancer mortality, as well as the link between low omega-3 intake and increased mortality from esophageal and gastric cancer. These insights can inform and guide policymakers and health officials toward strategies to reduce the impact of dietary risk factors on cancer death rates. However, further research is needed to fully understand the role of dietary factors in preventing or reducing cancer deaths.

## Data Availability

All the data analyzed during this study are included in this published article. For further inquiries regarding the data, the corresponding author was contacted.

## Abbreviations

PAF: Population Attributable fraction
WHO: World health organization
GBD: Global burden of disease
GCS: Golestan cohort study
GI: Gastrointestinal
N: Number
FFQ: Food frequency questionnaire
WCRF: World cancer research fund
HR: Hazard ratio
CI: Confidence interval
SES: Socioeconomic status
HEI: Healthy eating index
SD: Standard deviation
DASH: Dietary approach to stop hypertension
IQR: Interquartile range
